# 1-(2-Bromo-4-chloro­phen­yl)-3,3-di­methyl­thio­urea

**DOI:** 10.1107/S1600536814011350

**Published:** 2014-05-24

**Authors:** Gamal A. El-Hiti, Keith Smith, Amany S. Hegazy, Mohammad Hayal Alotaibi, Benson M. Kariuki

**Affiliations:** a Cornea Research Chair, Department of Optometry, College of Applied Medical Sciences, King Saud University, PO Box 10219, Riyadh 11433, Saudi Arabia; b School of Chemistry, Cardiff University, Main Building, Park Place, Cardiff CF10 3AT, Wales; c Petrochemical Research Institute, King Abdulaziz City for Science and Technology, PO Box 6086, Riyadh 11442, Saudi Arabia

## Abstract

In the title compound, C_9_H_10_BrClN_2_S, the di­methyl­thio­urea group is twisted from the benzene ring plane by 54.38 (6)°. In the crystal, the amino groups are involved in the formation of N—H⋯S hydrogen bonds, which link the mol­ecules into chains along [010]. Weak C—H⋯Cl inter­actions further link these chains into layers parallel to the *ab* plane.

## Related literature   

For related compounds, see: Maddani & Prabhu (2010 ▶); Yahyaza­deh & Ghasemi (2013 ▶); Zhao *et al.* (2013 ▶). For convenient routes for modifying urea derivatives *via* organolithium inter­mediates, see: Smith *et al.* (1996 ▶, 1999 ▶, 2009 ▶, 2010 ▶, 2012 ▶, 2014 ▶). For the structures of related compounds, see: Zhao *et al.* (2008 ▶); Ramnathan *et al.* (1996 ▶).
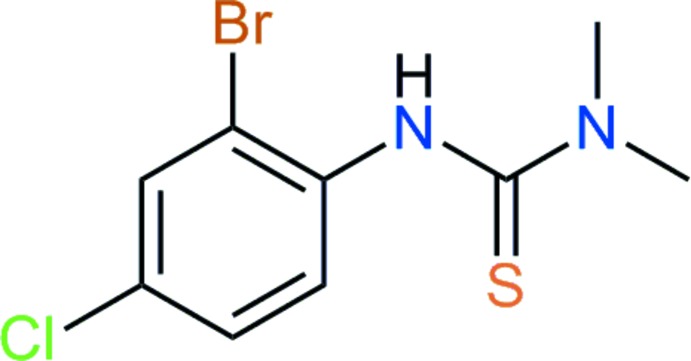


## Experimental   

### 

#### Crystal data  

C_9_H_10_BrClN_2_S
*M* *_r_* = 293.61
Monoclinic, 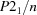

*a* = 12.1369 (3) Å
*b* = 7.9431 (2) Å
*c* = 13.2230 (4) Å
β = 115.386 (3)°
*V* = 1151.67 (6) Å^3^
*Z* = 4
Cu *K*α radiation
μ = 8.40 mm^−1^
*T* = 296 K
0.28 × 0.20 × 0.09 mm

#### Data collection  

Agilent SuperNova (Dual, Cu at zero, Atlas) diffractometer
Absorption correction: multi-scan (*CrysAlis PRO*; Agilent, 2014 ▶) *T*_min_ = 0.580, *T*_max_ = 1.000
4291 measured reflections
2245 independent reflections
2078 reflections with *I* > 2σ(*I*)
*R*_int_ = 0.015

#### Refinement  

*R*[*F* ^2^ > 2σ(*F* ^2^)] = 0.028
*wR*(*F* ^2^) = 0.077
*S* = 1.04
2245 reflections
130 parameters
H-atom parameters constrained
Δρ_max_ = 0.33 e Å^−3^
Δρ_min_ = −0.39 e Å^−3^

### 

Data collection: *CrysAlis PRO* (Agilent, 2014 ▶); cell refinement: *CrysAlis PRO*; data reduction: *CrysAlis PRO*; program(s) used to solve structure: *SHELXS2013* (Sheldrick, 2008 ▶); program(s) used to refine structure: *SHELXL2013* (Sheldrick, 2008 ▶); molecular graphics: *ORTEP-3 for Windows* (Farrugia, 2012 ▶); software used to prepare material for publication: *WinGX* (Farrugia, 2012 ▶).

## Supplementary Material

Crystal structure: contains datablock(s) I, New_Global_Publ_Block. DOI: 10.1107/S1600536814011350/cv5457sup1.cif

Structure factors: contains datablock(s) I. DOI: 10.1107/S1600536814011350/cv5457Isup2.hkl

Supporting information file. DOI: 10.1107/S1600536814011350/cv5457Isup3.cml

CCDC reference: 1003616

Additional supporting information:  crystallographic information; 3D view; checkCIF report

## Figures and Tables

**Table 1:** Hydrogen-bond geometry (Å, °)

*D*—H⋯*A*	*D*—H	H⋯*A*	*D*⋯*A*	*D*—H⋯*A*
N1—H1⋯S1^i^	0.86	2.67	3.349 (2)	137
C9—H9*B*⋯Cl1^ii^	0.96	2.81	3.696 (2)	153

Symmetry codes: (i) 
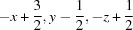
; (ii) 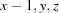.

**Fig. 1.**
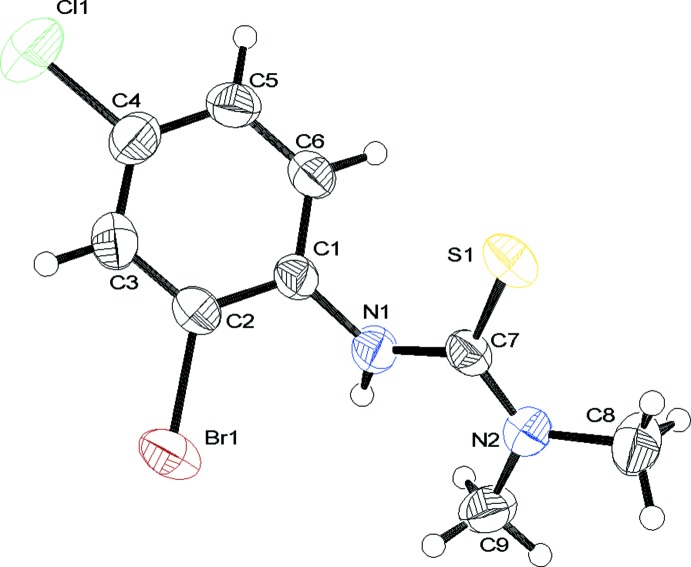
View of (I) showing atom labels and 50% probability displacement ellipsoids for non-H atoms.

**Fig. 2.**
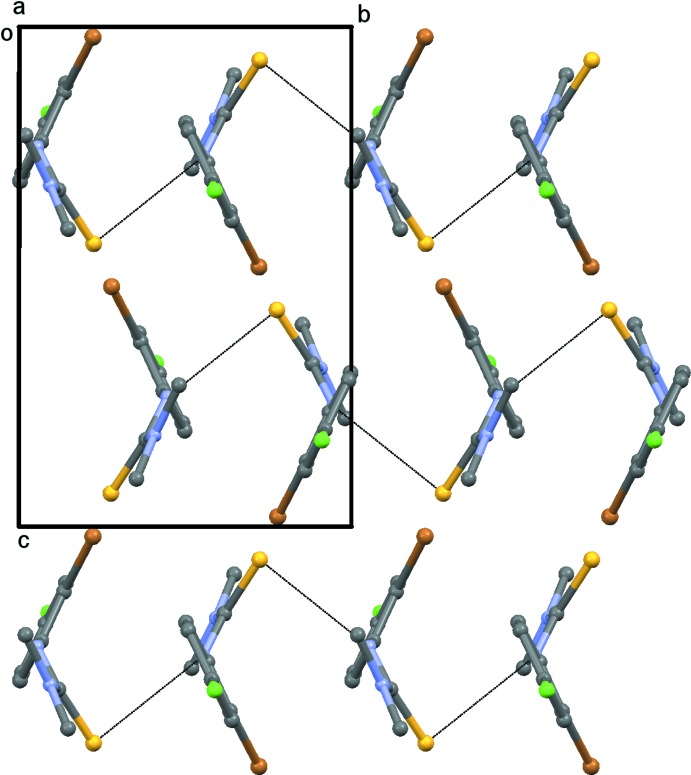
A portion of the crystal packing viewed along the a axis. N—H···S contacts are shown as dotted lines.

## supplementary crystallographic information

### 2. Structural commentary

Recently, various thio­urea derivatives have been synthesised and showed broad inter­esting properties (Maddani & Prabhu, 2010; Yahyaza­deh & Ghasemi, 2013; Zhao *et al.*, 2013). In a continuation of our research focused on new synthetic routes towards novel substituted urea derivatives (Smith *et al.*, 1996, 1999, 2009, 2010, 2012, 2014) we have synthesized 3-(2-bromo-4-chloro­phenyl)-1,1-di­methyl­thio­urea (I) in a high yield (Smith *et al*., 1996). We have prepared the material again and crystallized it in high purity in order to obtain its crystal structure, which we present herein.

In (I) (Fig. 1), all bond lengths and angles are normal and correspond well to those observed in the related compounds (Zhao *et al.*, 2008; Ramnathan *et al.*, 1996). The non-hydrogen atoms in (I) fall into two planes with an inter­planer angle of 54.38 (6)° between the bromo-chloro­phenyl and di­methyl­thio­urea groups. Each molecule is involved in N—H···S contacts (Table 1) with two neigbouring molecules, with one as an acceptor and the other as a donor, leading to the formation of zig-zag-chains in [010] (Fig 2). The bromo-chloro­phenyl and di­methyl­thio­urea groups of adjacent molecules are parallel in the stack forming chains of alternating S···Br···S groups with a separation of 4.07Å and 4.11Å between the atoms.

### 5. Synthesis and crystallization

To a stirred solution of 2-bromo-4-chloro-1-iso­thio­cyanato­benzene (12.43 g, 50.0 mmol) in anhydrous dioxane (120 ml) di­methyl­amine (7.10 g of 33% solution in ethanol, 52.0 mmol) was slowly added in a drop-wise manner over 5 min. The reaction mixture was stirred at room temperature for an extra 1 h. The solid obtained was collected by filtration and washed with dioxane (2 x 20 ml) and dried. Recrystallization from ethyl acetate gave 3-(2-bromo-4-chloro­phenyl)-1,1-di­methyl­thio­urea (13.80 g, 47.0 mmol; 94%) as yellow crystals, m.p. 193–194 °C [lit. 184–185 °C (ethyl acetate); Smith *et al.* (1996)]. ^1^H NMR (500 MHz, CDCl_3_, *δ*, p.p.m.) 7.97 (d, *J* = 8.8 Hz, 1 H, H-6), 7.59 (d, *J* = 2.3 Hz, 1 H, H-3), 7.32 (dd, *J* = 2.3, 8.8 Hz, 1 H, H-5), 7.17 (br, exch., 1 H, NH), 3.43 [s, 6 H, N(CH_3_)_2_]. ^13^C NMR (125 MHz, CDCl_3_, *δ*, p.p.m.) 181.2 (s, C=S), 136.6 (s, C-1), 131.8 (d, C-3), 131.0 (s, C-4), 127.8 (d, C-6), 127.7 (d, C-5), 118.1 (s, C-2), 41.3 [q, N(CH_3_)_2_]. AP^+^—MS (*m/z*, %): 297 ([*MH*^81^Br^37^Cl]^+^, 34), 295 ([*MH*^81^Br^35^Cl and *MH*^79^Br^37^Cl]^+^, 100), 293 ([*MH*^79^Br^35^Cl]^+^, 80), 263 (12), 215 (22), 213 (50). HRMS (AP^+^): Calculated for C_9_H_11_^79^Br^35^ClN_2_S [*MH*] 292.9515; found, 292.9515.

### 6. Refinement

H atoms were positioned geometrically and refined using a riding model with *U*ĩso(H) = 1.2 times *U*eq for the atom they are bonded to except for the methyl groups where 1.5 times *U*eq was used with free rotation about the C—C bond.

### Crystal data

**Table:**

C_9_H_10_BrClN_2_S	*F*(000) = 584
*M**_r_* = 293.61	*D*_x_ = 1.693 Mg m^−^^3^
Monoclinic, *P*2_1_/*n*	Cu *K*α radiation, λ = 1.5418 Å
*a* = 12.1369 (3) Å	Cell parameters from 2078 reflections
*b* = 7.9431 (2) Å	θ = 4.1–75.5°
*c* = 13.2230 (4) Å	µ = 8.40 mm^−^^1^
β = 115.386 (3)°	*T* = 296 K
*V* = 1151.67 (6) Å^3^	Plate, colourless
*Z* = 4	0.28 × 0.20 × 0.09 mm

### Data collection

**Table:**

Agilent SuperNova (Dual, Cu at zero, Atlas) diffractometer	2245 independent reflections
Radiation source: SuperNova (Cu) X-ray Source	2078 reflections with *I* > 2σ(*I*)
Mirror monochromator	*R*_int_ = 0.015
ω scans	θ_max_ = 73.5°, θ_min_ = 4.1°
Absorption correction: multi-scan (*CrysAlis PRO*; Agilent, 2014)	*h* = −13→14
*T*_min_ = 0.580, *T*_max_ = 1.000	*k* = −9→6
4291 measured reflections	*l* = −16→15

### Refinement

**Table:**

Refinement on *F*^2^	Hydrogen site location: inferred from neighbouring sites
Least-squares matrix: full	H-atom parameters constrained
*R*[*F*^2^ > 2σ(*F*^2^)] = 0.028	*w* = 1/[σ^2^(*F*_o_^2^) + (0.0429*P*)^2^ + 0.4682*P*] where *P* = (*F*_o_^2^ + 2*F*_c_^2^)/3
*wR*(*F*^2^) = 0.077	(Δ/σ)_max_ = 0.002
*S* = 1.04	Δρ_max_ = 0.33 e Å^−^^3^
2245 reflections	Δρ_min_ = −0.39 e Å^−^^3^
130 parameters	Extinction correction: *SHELXL2013* (Sheldrick, 2008), Fc^*^=kFc[1+0.001xFc^2^λ^3^/sin(2θ)]^-1/4^
0 restraints	Extinction coefficient: 0.0048 (3)

### Special details

**Table:**

Experimental. Absorption correction: CrysAlisPro (Agilent, 2014). Empirical absorption correction using spherical harmonics, implemented in SCALE3 ABSPACK scaling algorithm.
Geometry. All e.s.d.'s (except the e.s.d. in the dihedral angle between two l.s. planes) are estimated using the full covariance matrix. The cell e.s.d.'s are taken into account individually in the estimation of e.s.d.'s in distances, angles and torsion angles; correlations between e.s.d.'s in cell parameters are only used when they are defined by crystal symmetry. An approximate (isotropic) treatment of cell e.s.d.'s is used for estimating e.s.d.'s involving l.s. planes.

### Fractional atomic coordinates and isotropic or equivalent isotropic displacement parameters (Å^2^)

**Table:**

	*x*	*y*	*z*	*U*_iso_*/*U*_eq_	
C1	0.85876 (18)	0.0661 (3)	0.22866 (16)	0.0377 (4)	
C2	0.85637 (19)	0.1344 (3)	0.13058 (16)	0.0404 (4)	
C3	0.9605 (2)	0.1405 (3)	0.11271 (19)	0.0495 (5)	
H3	0.9581	0.1851	0.0468	0.059*	
C4	1.0675 (2)	0.0794 (3)	0.1942 (2)	0.0523 (5)	
C5	1.0726 (2)	0.0087 (3)	0.29241 (19)	0.0497 (5)	
H5	1.1455	−0.0334	0.3465	0.060*	
C6	0.96763 (19)	0.0022 (3)	0.30797 (17)	0.0440 (5)	
H6	0.9699	−0.0460	0.3729	0.053*	
C7	0.72784 (18)	0.1177 (3)	0.32603 (16)	0.0379 (4)	
C8	0.5801 (3)	0.1471 (4)	0.4043 (2)	0.0638 (7)	
H8A	0.5786	0.0517	0.4483	0.096*	
H8B	0.5006	0.1978	0.3710	0.096*	
H8C	0.6383	0.2278	0.4515	0.096*	
C9	0.5176 (2)	0.0190 (4)	0.2169 (2)	0.0569 (6)	
H9A	0.5032	0.0890	0.1533	0.085*	
H9B	0.4443	0.0110	0.2274	0.085*	
H9C	0.5416	−0.0913	0.2045	0.085*	
Br1	0.70976 (2)	0.22178 (4)	0.01974 (2)	0.05768 (14)	
Cl1	1.19895 (7)	0.08827 (15)	0.17270 (8)	0.0936 (3)	
N1	0.74917 (15)	0.0536 (3)	0.24110 (14)	0.0442 (4)	
H1	0.6901	−0.0001	0.1897	0.053*	
N2	0.61441 (17)	0.0925 (3)	0.31655 (16)	0.0470 (4)	
S1	0.83592 (5)	0.22395 (7)	0.43366 (4)	0.04637 (16)	

### Atomic displacement parameters (Å^2^)

**Table:**

	*U*^11^	*U*^22^	*U*^33^	*U*^12^	*U*^13^	*U*^23^
C1	0.0377 (9)	0.0420 (10)	0.0314 (9)	−0.0038 (8)	0.0129 (7)	−0.0039 (8)
C2	0.0436 (10)	0.0403 (10)	0.0335 (9)	−0.0009 (8)	0.0128 (8)	−0.0013 (8)
C3	0.0570 (13)	0.0531 (13)	0.0436 (11)	−0.0046 (10)	0.0264 (10)	0.0013 (10)
C4	0.0437 (11)	0.0644 (15)	0.0526 (12)	−0.0043 (10)	0.0242 (10)	−0.0078 (11)
C5	0.0395 (10)	0.0595 (14)	0.0420 (11)	0.0018 (9)	0.0098 (9)	−0.0050 (10)
C6	0.0451 (10)	0.0495 (12)	0.0327 (9)	0.0004 (9)	0.0123 (8)	0.0013 (8)
C7	0.0420 (10)	0.0375 (10)	0.0312 (9)	0.0047 (8)	0.0128 (8)	0.0048 (7)
C8	0.0684 (15)	0.0766 (18)	0.0611 (15)	0.0111 (14)	0.0416 (13)	0.0006 (13)
C9	0.0376 (10)	0.0679 (16)	0.0603 (14)	0.0018 (10)	0.0164 (10)	−0.0056 (12)
Br1	0.05856 (19)	0.0600 (2)	0.04035 (17)	0.01302 (11)	0.00771 (12)	0.00683 (10)
Cl1	0.0558 (4)	0.1432 (9)	0.0976 (6)	−0.0020 (4)	0.0480 (4)	−0.0011 (6)
N1	0.0376 (8)	0.0595 (11)	0.0339 (8)	−0.0088 (8)	0.0138 (7)	−0.0088 (8)
N2	0.0435 (9)	0.0547 (11)	0.0451 (9)	0.0054 (8)	0.0213 (8)	−0.0008 (8)
S1	0.0545 (3)	0.0447 (3)	0.0308 (3)	0.0000 (2)	0.0096 (2)	−0.00197 (19)

### Geometric parameters (Å, º)

**Table:**

C1—C6	1.384 (3)	C7—N2	1.343 (3)
C1—C2	1.395 (3)	C7—N1	1.355 (3)
C1—N1	1.412 (3)	C7—S1	1.690 (2)
C2—C3	1.383 (3)	C8—N2	1.457 (3)
C2—Br1	1.887 (2)	C8—H8A	0.9600
C3—C4	1.372 (3)	C8—H8B	0.9600
C3—H3	0.9300	C8—H8C	0.9600
C4—C5	1.392 (3)	C9—N2	1.459 (3)
C4—Cl1	1.738 (2)	C9—H9A	0.9600
C5—C6	1.375 (3)	C9—H9B	0.9600
C5—H5	0.9300	C9—H9C	0.9600
C6—H6	0.9300	N1—H1	0.8600
			
C6—C1—C2	118.68 (18)	N1—C7—S1	122.03 (16)
C6—C1—N1	121.79 (18)	N2—C8—H8A	109.5
C2—C1—N1	119.39 (18)	N2—C8—H8B	109.5
C3—C2—C1	121.09 (19)	H8A—C8—H8B	109.5
C3—C2—Br1	118.76 (16)	N2—C8—H8C	109.5
C1—C2—Br1	120.14 (15)	H8A—C8—H8C	109.5
C4—C3—C2	118.7 (2)	H8B—C8—H8C	109.5
C4—C3—H3	120.7	N2—C9—H9A	109.5
C2—C3—H3	120.7	N2—C9—H9B	109.5
C3—C4—C5	121.6 (2)	H9A—C9—H9B	109.5
C3—C4—Cl1	118.89 (19)	N2—C9—H9C	109.5
C5—C4—Cl1	119.51 (19)	H9A—C9—H9C	109.5
C6—C5—C4	118.8 (2)	H9B—C9—H9C	109.5
C6—C5—H5	120.6	C7—N1—C1	126.45 (17)
C4—C5—H5	120.6	C7—N1—H1	116.8
C5—C6—C1	121.2 (2)	C1—N1—H1	116.8
C5—C6—H6	119.4	C7—N2—C8	120.9 (2)
C1—C6—H6	119.4	C7—N2—C9	122.72 (18)
N2—C7—N1	114.77 (18)	C8—N2—C9	116.3 (2)
N2—C7—S1	123.19 (16)		

### Hydrogen-bond geometry (Å, º)

**Table:**

*D*—H···*A*	*D*—H	H···*A*	*D*···*A*	*D*—H···*A*
N1—H1···S1^i^	0.86	2.67	3.3488 (19)	137
C9—H9*B*···Cl1^ii^	0.96	2.81	3.696 (2)	153

Symmetry codes: (i) −*x*+3/2, *y*−1/2, −*z*+1/2; (ii) *x*−1, *y*, *z*.
